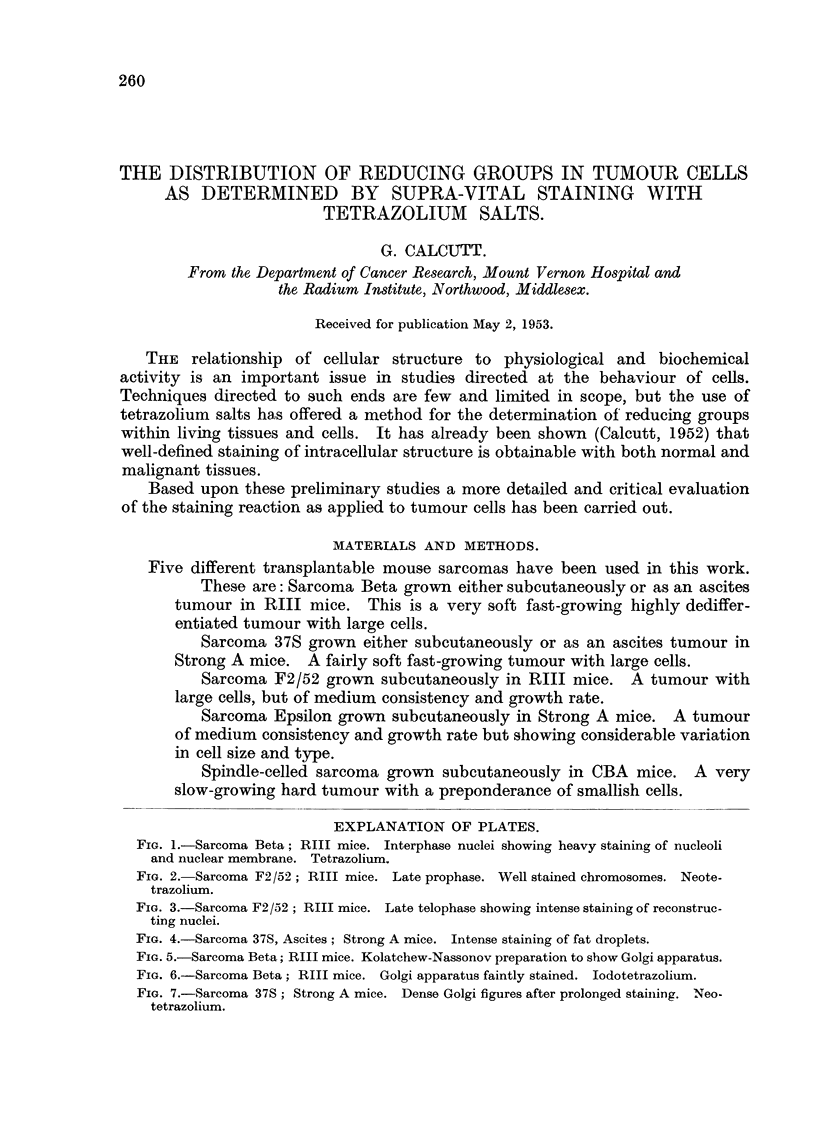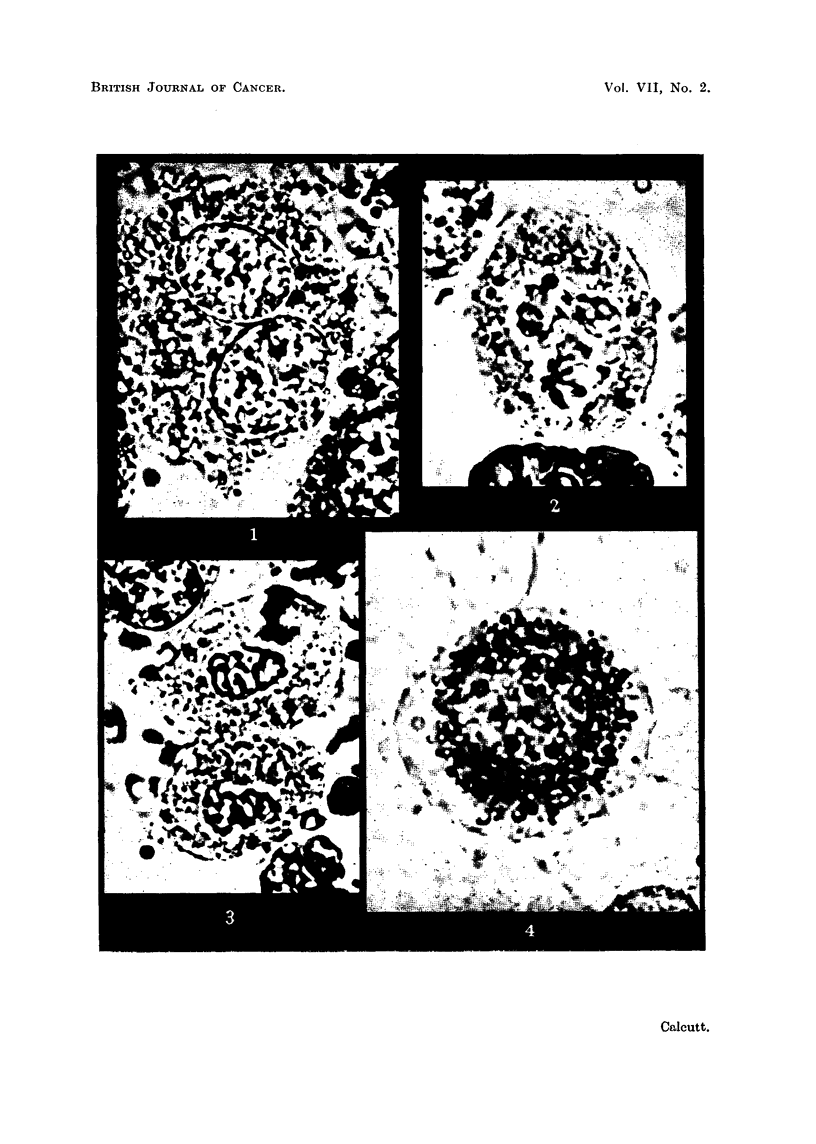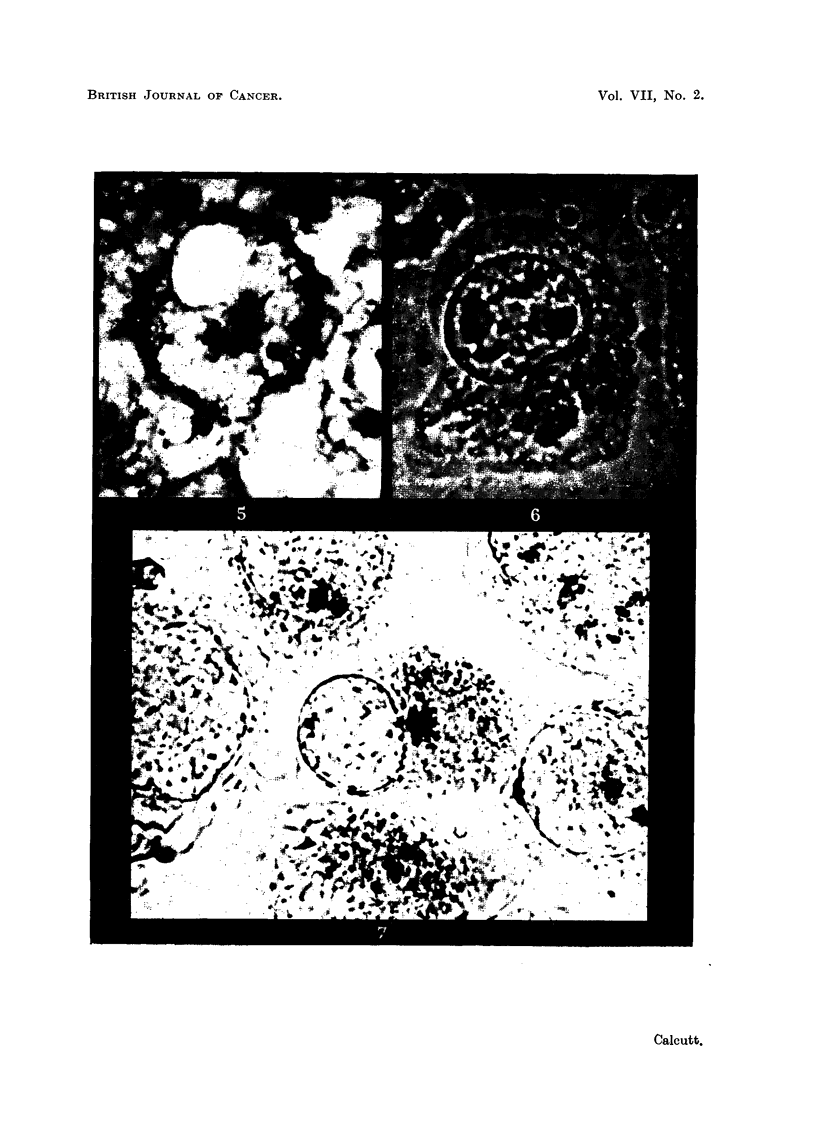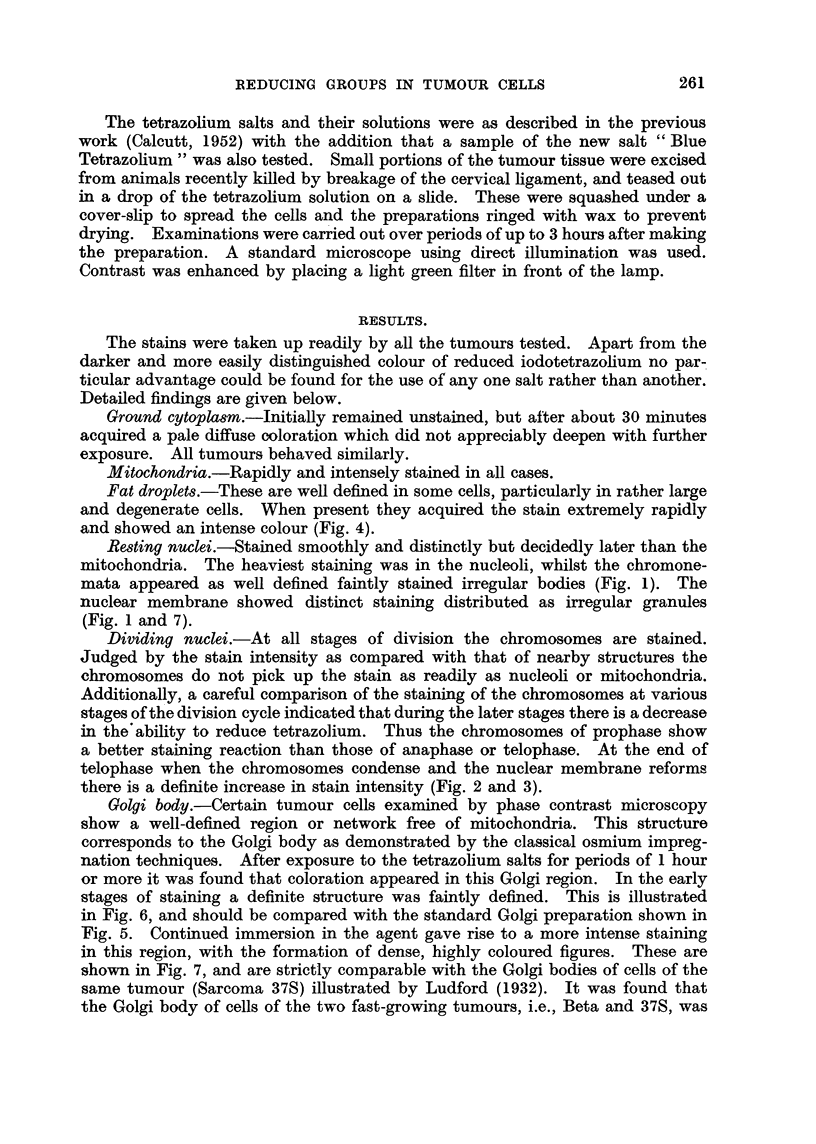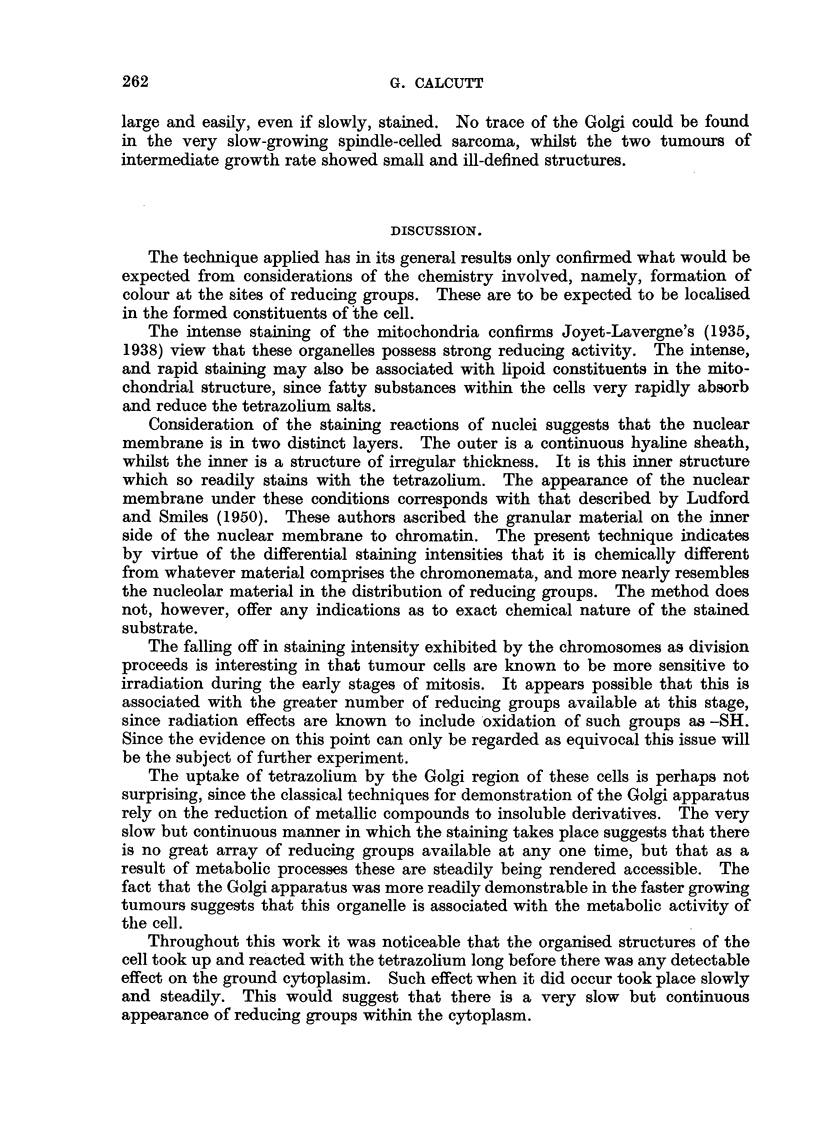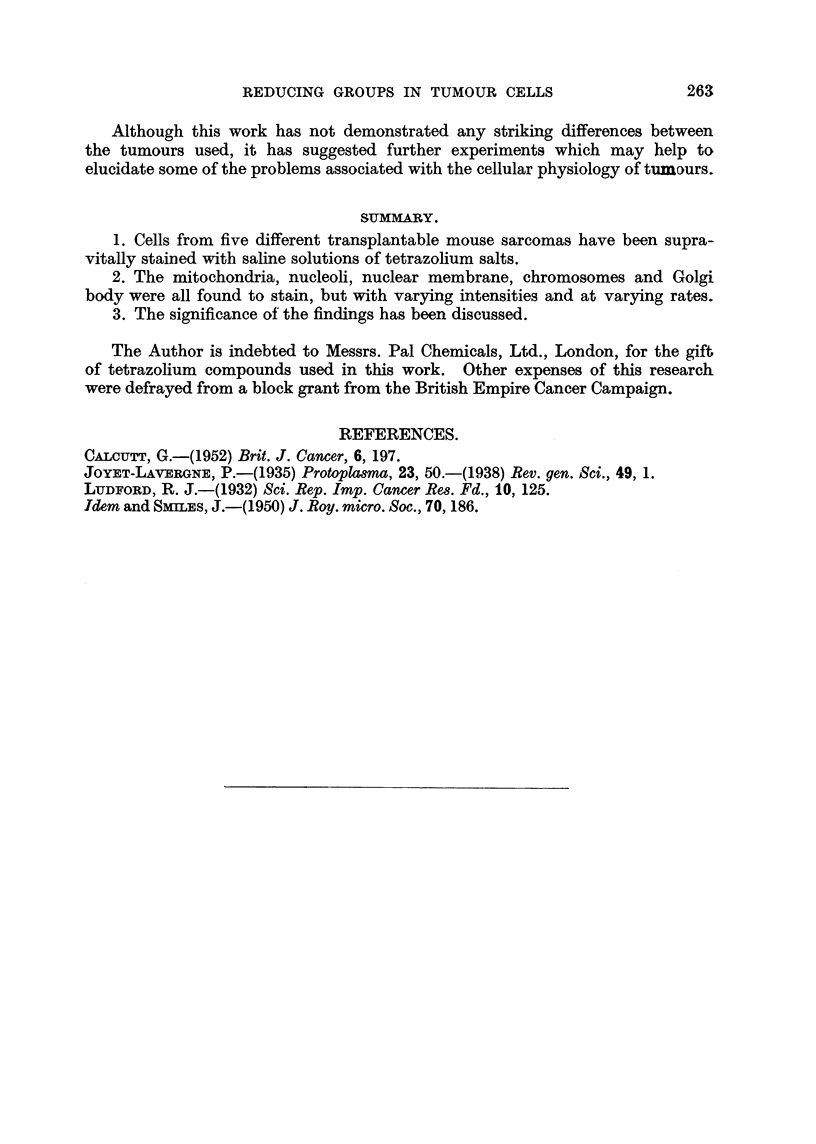# The Distribution of Reducing Groups in Tumour Cells as Determined by Supra-Vital Staining with Tetrazolium Salts

**DOI:** 10.1038/bjc.1953.24

**Published:** 1953-06

**Authors:** G. Calcutt

## Abstract

**Images:**


### THE DISTRIBUTION OF REDUCING GROUPS IN TUMOUR CELLS AS DETERMINED BY SUPRA-VITAL STAINING WITH TETRAZOLIUM SALTS.

G. CALCUTT.

*From the Department of Cancer Research, Mount Vernon Hospital and the Radium Institute, Northwood, Middlesex.*

Received for publication May 2, 1953.

THE relationship of cellular structure to physiological and biochemical activity is an important issue in studies directed at the behaviour of cells. Techniques directed to such ends are few and limited in scope, but the use of tetrazolium salts has offered a method for the determination of reducing groups within living tissues and cells. It has already been shown (Calcutt, 1952) that well-defined staining of intracellular structure is obtainable with both normal and malignant tissues.

Based upon these preliminary studies a more detailed and critical evaluation of the staining reaction as applied to tumour cells has been carried out.

### MATERIALS AND METHODS.

Five different transplantable mouse sarcomas have been used in this work.

These are: Sarcoma Beta grown either subcutaneously or as an ascites tumour in RIII mice. This is a very soft fast-growing highly dedifferentiated tumour with large cells.

Sarcoma 37S grown either subcutaneously or as an ascites tumour in Strong A mice. A fairly soft fast-growing tumour with large cells.

Sarcoma F2/52 grown subcutaneously in RIII mice. A tumour with large cells, but of medium consistency and growth rate.

Sarcoma Epsilon grown subcutaneously in Strong A mice. A tumour of medium consistency and growth rate but showing considerable variation in cell size and type.

Spindle-celled sarcoma grown subcutaneously in CBA mice. A very slow-growing hard tumour with a preponderance of smallish cells.

---

EXPLANATION OF PLATES.

FIG. 1.—Sarcoma Beta ; RIII mice. Interphase nuclei showing heavy staining of nucleoli and nuclear membrane. Tetrazolium.

FIG. 2.—Sarcoma F2/52 ; RIII mice. Late prophase. Well stained chromosomes. Neotetrazolium.

FIG. 3.—Sarcoma F2/52 ; RIII mice. Late telophase showing intense staining of reconstructing nuclei.

FIG. 4.—Sarcoma 37S, Ascites ; Strong A mice. Intense staining of fat droplets.

FIG. 5.—Sarcoma Beta ; RIII mice. Kolatchew-Nassonov preparation to show Golgi apparatus.

FIG. 6.—Sarcoma Beta ; RIII mice. Golgi apparatus faintly stained. Iodotetrazolium.

FIG. 7.—Sarcoma 37S ; Strong A mice. Dense Golgi figures after prolonged staining. Neotetrazolium.

Calcutt.

Calcutt.

The tetrazolium salts and their solutions were as described in the previous work (Calcutt, 1952) with the addition that a sample of the new salt " Blue Tetrazolium " was also tested. Small portions of the tumour tissue were excised from animals recently killed by breakage of the cervical ligament, and teased out in a drop of the tetrazolium solution on a slide. These were squashed under a cover-slip to spread the cells and the preparations ringed with wax to prevent drying. Examinations were carried out over periods of up to 3 hours after making the preparation. A standard microscope using direct illumination was used. Contrast was enhanced by placing a light green filter in front of the lamp.

### RESULTS.

The stains were taken up readily by all the tumours tested. Apart from the darker and more easily distinguished colour of reduced iodotetrazolium no particular advantage could be found for the use of any one salt rather than another. Detailed findings are given below.

*Ground cytoplasm.*—Initially remained unstained, but after about 30 minutes acquired a pale diffuse coloration which did not appreciably deepen with further exposure. All tumours behaved similarly.

*Mitochondria.*—Rapidly and intensely stained in all cases.

*Fat droplets.*—These are well defined in some cells, particularly in rather large and degenerate cells. When present they acquired the stain extremely rapidly and showed an intense colour (Fig. 4).

*Resting nuclei.*—Stained smoothly and distinctly but decidedly later than the mitochondria. The heaviest staining was in the nucleoli, whilst the chromonemata appeared as well defined faintly stained irregular bodies (Fig. 1). The nuclear membrane showed distinct staining distributed as irregular granules (Fig. 1 and 7).

*Dividing nuclei.*—At all stages of division the chromosomes are stained. Judged by the stain intensity as compared with that of nearby structures the chromosomes do not pick up the stain as readily as nucleoli or mitochondria. Additionally, a careful comparison of the staining of the chromosomes at various stages of the division cycle indicated that during the later stages there is a decrease in the ability to reduce tetrazolium. Thus the chromosomes of prophase show a better staining reaction than those of anaphase or telophase. At the end of telophase when the chromosomes condense and the nuclear membrane reforms there is a definite increase in stain intensity (Fig. 2 and 3).

*Golgi body.*—Certain tumour cells examined by phase contrast microscopy show a well-defined region or network free of mitochondria. This structure corresponds to the Golgi body as demonstrated by the classical osmium impregnation techniques. After exposure to the tetrazolium salts for periods of 1 hour or more it was found that coloration appeared in this Golgi region. In the early stages of staining a definite structure was faintly defined. This is illustrated in Fig. 6, and should be compared with the standard Golgi preparation shown in Fig. 5. Continued immersion in the agent gave rise to a more intense staining in this region, with the formation of dense, highly coloured figures. These are shown in Fig. 7, and are strictly comparable with the Golgi bodies of cells of the same tumour (Sarcoma 37S) illustrated by Ludford (1932). It was found that the Golgi body of cells of the two fast-growing tumours, i.e., Beta and 37S, was large and easily, even if slowly, stained. No trace of the Golgi could be found in the very slow-growing spindle-celled sarcoma, whilst the two tumours of intermediate growth rate showed small and ill-defined structures.

### DISCUSSION.

The technique applied has in its general results only confirmed what would be expected from considerations of the chemistry involved, namely, formation of colour at the sites of reducing groups. These are to be expected to be localised in the formed constituents of the cell.

The intense staining of the mitochondria confirms Joyet-Lavergne's (1935, 1938) view that these organelles possess strong reducing activity. The intense, and rapid staining may also be associated with lipoid constituents in the mitochondrial structure, since fatty substances within the cells very rapidly absorb and reduce the tetrazolium salts.

Consideration of the staining reactions of nuclei suggests that the nuclear membrane is in two distinct layers. The outer is a continuous hyaline sheath, whilst the inner is a structure of irregular thickness. It is this inner structure which so readily stains with the tetrazolium. The appearance of the nuclear membrane under these conditions corresponds with that described by Ludford and Smiles (1950). These authors ascribed the granular material on the inner side of the nuclear membrane to chromatin. The present technique indicates by virtue of the differential staining intensities that it is chemically different from whatever material comprises the chromonemata, and more nearly resembles the nucleolar material in the distribution of reducing groups. The method does not, however, offer any indications as to exact chemical nature of the stained substrate.

The falling off in staining intensity exhibited by the chromosomes as division proceeds is interesting in that tumour cells are known to be more sensitive to irradiation during the early stages of mitosis. It appears possible that this is associated with the greater number of reducing groups available at this stage, since radiation effects are known to include oxidation of such groups as –SH. Since the evidence on this point can only be regarded as equivocal this issue will be the subject of further experiment.

The uptake of tetrazolium by the Golgi region of these cells is perhaps not surprising, since the classical techniques for demonstration of the Golgi apparatus rely on the reduction of metallic compounds to insoluble derivatives. The very slow but continuous manner in which the staining takes place suggests that there is no great array of reducing groups available at any one time, but that as a result of metabolic processes these are steadily being rendered accessible. The fact that the Golgi apparatus was more readily demonstrable in the faster growing tumours suggests that this organelle is associated with the metabolic activity of the cell.

Throughout this work it was noticeable that the organised structures of the cell took up and reacted with the tetrazolium long before there was any detectable effect on the ground cytoplasim. Such effect when it did occur took place slowly and steadily. This would suggest that there is a very slow but continuous appearance of reducing groups within the cytoplasm.

Although this work has not demonstrated any striking differences between the tumours used, it has suggested further experiments which may help to elucidate some of the problems associated with the cellular physiology of tumours.

SUMMARY.

1. Cells from five different transplantable mouse sarcomas have been supravitally stained with saline solutions of tetrazolium salts.
2. The mitochondria, nucleoli, nuclear membrane, chromosomes and Golgi body were all found to stain, but with varying intensities and at varying rates.
3. The significance of the findings has been discussed.

The Author is indebted to Messrs. Pal Chemicals, Ltd., London, for the gift of tetrazolium compounds used in this work. Other expenses of this research were defrayed from a block grant from the British Empire Cancer Campaign.

### REFERENCES.

CALCUTT, G.—(1952) *Brit. J. Cancer*, **6**, 197.
JOYET-LAVERGNE, P.—(1935) *Protoplasma*, **23**, 50.—(1938) *Rev. gen. Sci.*, **49**, 1.
LUDFORD, R. J.—(1932) *Sci. Rep. Imp. Cancer Res. Fd.*, **10**, 125.
*Idem* and SMILES, J.—(1950) *J. Roy. micro. Soc.*, **70**, 186.